# Intrauterine Infusion of Human Platelet-Rich Plasma Improves Endometrial Regeneration and Pregnancy Outcomes in a Murine Model of Asherman’s Syndrome

**DOI:** 10.3389/fphys.2020.00105

**Published:** 2020-02-14

**Authors:** Ji Hye Kim, Mira Park, Jin Young Paek, Woo-Sik Lee, Haengseok Song, Sang Woo Lyu

**Affiliations:** ^1^Department of Obstetrics and Gynecology, Fertility Center of CHA Gangnam Medical Center, CHA University, Seoul, South Korea; ^2^Department of Biomedical Science, CHA University, Seongnam, South Korea; ^3^Department of Laboratory Medicine, CHA Gangnam Medical Center, CHA University, Seoul, South Korea

**Keywords:** platelet-rich plasma, Asherman’s syndrome, thin endometrium, regeneration, pregnancy outcome

## Abstract

Asherman’s syndrome (AS) is characterized by intrauterine adhesion or fibrosis resulting from damage to the endometrium, often leading to amenorrhea, infertility, or recurrent pregnancy loss. Although various therapeutic strategies for AS have been proposed, the options remain limited. New strategies such as bone marrow-derived mesenchymal stem cell (BM-MSC) therapy aim to potentiate the intrinsic capacity of endometrial regeneration. However, BM-MSC therapy has not been widely adopted mainly because it involves invasive and expensive procedures such as bone marrow biopsy and cell storing. On the other hand, platelet-rich plasma (PRP) is considered safe and affordable because it involves the less invasive procedure of blood collection from peripheral veins to produce PRP. To assess the effectiveness of human PRP infusion for endometrial regeneration, we established a murine model of injury-induced AS and evaluated endometrial morphology, expression of fibrosis-related factors, implantation sites (IS), and pregnancy outcomes associated with human PRP treatment. We found that treatment with human PRP was associated with improved endometrial morphology, reduced degree of fibrosis, and down-regulated expression of fibrosis-related factors in murine model of AS. Furthermore, human PRP treatment was associated with a higher number of IS and live-births. Our results suggest that human PRP treatment may become a valuable strategy to promote the regeneration of damaged endometrium and thus improve fertility and pregnancy outcomes in clinical practice.

## Introduction

The human endometrium undergoes cyclic regeneration and breakdown during the reproductive years of a woman’s life (Chan and Gargett, 2006; Masuda et al., 2010). The endometrium is composed of an upper functional layer and a lower basal layer. The basal layer is the source of endometrial reconstruction, giving rise to the new functional layer at monthly intervals (Chan and Gargett, 2006). Specifically, in each menstruation cycle, the superficial functional layer of the endometrium is shed and reconstituted out of the underlying basal layer.

In Asherman’s syndrome (AS), injury to the endometrium due to repeated or aggressive curettage or endometritis causes dysfunction of the endometrial regeneration process, resulting in loss of functional endometrium and obliteration of the uterine cavity due to intrauterine adhesion (Cervello et al., 2015). Women with atrophic and/or fibrotic endometrium have menstrual abnormalities, especially amenorrhea, and impaired fertility manifesting as, reduced pregnancy rates, poor pregnancy outcomes, and recurrent pregnancy loss (Conforti et al., 2013). Current treatments for AS aim to improve endometrial regeneration by using exogenous estrogen, low-dose aspirin, vaginal sildenafil citrate, pentoxifylline, vitamin E, L-arginine, colony-stimulating factor, and cytokines; however, such strategies have had limited success (Senturk and Erel, 2008; Conforti et al., 2013; Cervello et al., 2015). Recently, there has been a growing interest in developing mesenchymal stem cell (MSC) therapy for refractory endometrium, as the presence of endometrial stem cells in the human endometrium is thought to play a role in endometrial regeneration (Mouhayar and Sharara, 2017). In particular, bone marrow-derived MSC (BM-MSC) therapy has been shown to promote the reconstruction of murine and human endometrium (Gargett and Healy, 2011; Cervello et al., 2015; Mouhayar and Sharara, 2017). While various reports support the use of stem-cell therapy in AS patients and refractory to other treatment, stem-cell therapy has not been widely adopted in clinical practice mainly because it involves very invasive and expensive procedures such as bone marrow biopsy and cell storing (Mouhayar and Sharara, 2017). It is desirable to identify alternative treatment strategies that are safe, cost effective, and convenient for the patients.

Platelet-rich plasma (PRP) infusion can promote tissue regeneration in patients with various clinical conditions including ulcers, burns, muscle damage, and bone disease, as well as in patients recovering after surgery (Etulain et al., 2018). PRP is an autologous concentration of platelets in a small volume of plasma, containing more than 1,000,000 platelets per microliter (Jang et al., 2017). PRP releases several growth factors such as vascular endothelial growth factor, epidermal growth factor, platelet-derived growth factor, transforming growth factor (TGF), and other cytokines stored in the alpha granules of platelets (Lee et al., 2013; Chang et al., 2015; Zadehmodarres et al., 2017; Etulain et al., 2018). The released growth factors modulate angiogenesis, remodel the extracellular matrix and affect the recruitment proliferation, and differentiation of stem cells (De Pascale et al., 2015; Burnouf et al., 2016; Etulain et al., 2018). In contrast with other regenerative approaches such as BM-MSC therapy, the use of PRP is considered safe and affordable because it involves a less invasive procedure of collecting blood from peripheral veins to produce PRP (Amable et al., 2013; Chang et al., 2015; Jang et al., 2017; Etulain et al., 2018). Some reports and pilot studies suggested that intrauterine infusion of autologous PRP had a positive effect on endometrial growth and/or pregnancy outcomes in patients with impaired endometrial growth (Chang et al., 2015; Farimani et al., 2017; Tandulwadkar et al., 2017; Zadehmodarres et al., 2017; Kim et al., 2019). However, the scientific basis for the effect of human PRP has not been clarified, which impedes the widespread adoption of human PRP therapy for AS. Only one study applied molecular biological experiments to quantify the effect of endometrial regeneration after autologous PRP treatment in a rat model (Jang et al., 2017), but did not conduct functional analysis to elucidate the therapeutic role of PRP.

To evaluate the potential pharmacologic use of human PRP in the clinical setting, we developed a murine model of AS and conducted histological, biological, and functional analysis to investigate whether human PRP administration could restore endometrial function and improve pregnancy outcomes.

## Materials and Methods

### Study Design

This study was approved by the Institutional Review Board (IRB approval number, GCI-17-19) of the CHA Gangnam Medical Center. For PRP preparation, blood samples were obtained after the donors provided informed consent. All donors are Asian females with ages ranging from 33 to 45 years (average 38.1 ± 3.5 years). All experiments utilizing animals were approved by the Institutional Animal Care and Use Committee (IACUC, approval number, 170088) of CHA University. The animals were handled in strict accordance with the institutional recommendations and best practices on the care and use of laboratory animals. All efforts were made to minimize suffering.

**FIGURE 1:**
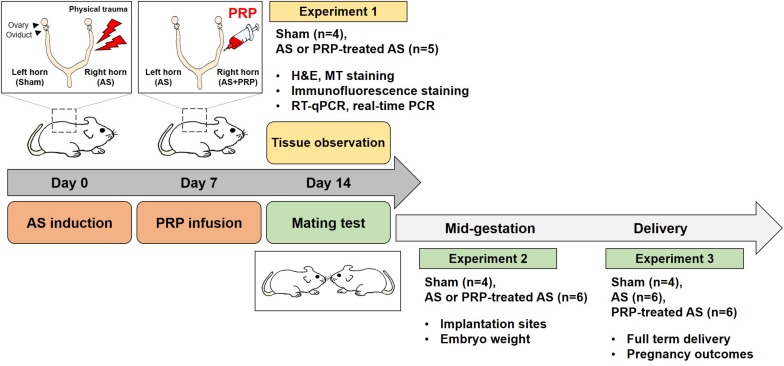
Experimental design. Three experiments were performed to examine the therapeutic effect of human PRP on endometrial regeneration. In Experiment 1, the mice were euthanized at 14 days after uterine horn damage (7 days after PRP treatment) and the effects on endometrial regeneration were assessed by evaluating endometrial histology and expression of fibrosis-related factors. In Experiments 2 and 3, the mice were mated at 14 days after experiment initiation (7 days after PRP treatment) and followed-up until mid-gestation and delivery, respectively. In Experiment 2, the mice were euthanized on day 12 of pregnancy to examine the implantation sites. In Experiment 3, the pregnancy outcomes were evaluated.

Three experiments were conducted, each with a different purpose: Experiment 1 to assess endometrial regeneration (endometrium histology, expression of fibrosis-related factors), Experiment 2 to evaluate implantation outcomes at mid-gestation [number of implantation sites (IS), embryo development, weight of IS], and Experiment 3 to evaluate pregnancy outcomes (time to conceive, live-birth rate, litter size). Three groups of 8-week old CB17 Prkdc^scid^/Ncrkoat female mice with severe combined immunodeficiency (SCID) were evaluated in all experiments: no uterine damage (Sham group), uterine damage with no PRP treatment (AS group), and uterine damage followed by PRP treatment (PRP-treated AS group). In AS mice (mice with AS induced by uterine damage) used for Experiments 1 and 2, uterine horn damage was induced bilaterally and human PRP was injected unilaterally (in the right horn) at 7 days after AS induction (uterine horn damage). Thus, data pertaining to AS mice from Experiments 1 and 2 were further stratified according to whether they were obtained for the PRP-treated AS horn (PRP-treated AS group) or for the untreated AS horn (AS group). In Experiment 3, some AS mice received bilateral uterine horn injury followed by bilateral PRP treatment (PRP-treated AS group) at 7 days after AS induction, whereas other AS mice received bilateral uterine horn injury with no PRP treatment (AS group). An overview of the study design is provided in Figure 1.

### Preparation of PRP

Platelet-rich plasma was used to observe the histological and molecular biological effects using animal models. Blood samples were obtained from six otherwise healthy female donors with AS, with no active infection. A 22 mL volume of whole blood was drawn from each donor using a syringe containing 3 mL of anticoagulant solution (citrate dextrose, ACD-A) and prepared using a PRS Bio Kit (PRODIZEN, Inc., Seoul, South Korea). The samples were centrifuged at 3,000 rpm for 5 min. After blood fractionation into three layers (plasma, buffy coat, and erythrocyte fraction), the plasma and buffy coat were separated. A syringe was used to extract approximately 0.5 mL of plasma and 0.2 mL of buffy coat (platelets and leukocytes), obtaining 0.7 mL of PRP. Of the total PRP volume collected for each donor, 150 μL was sent to the laboratory to confirm the platelet concentration. Among the samples harvested from the six donors, the mean concentration of platelets was 232,200 cells/μL in the original sample and 1,871,000 cells/μL in the PRP.

### Establishment of a Murine Model of AS

We used an optimized protocol to establish a murine model of injury-induced AS. CB17 *Prkdc*^*scid*^/Ncrkoat female mice with SCID were housed in the Animal Care Facility of CHA University, under temperature- and light-controlled conditions (12-h light/dark cycle), and fed *ad libitum*. SCID mice were used for human PRP therapy on AS because they allow allogeneic and xenogeneic grafts. Eight-week-old mice provided by KOATECH (Pyeongtaek, Gyeonggi, South Korea) were used to establish an AS murine model according to a previously published protocol, with slight modifications (Alawadhi et al., 2014; Jun et al., 2019). The mice were anesthetized via intraperitoneal injection of tribromoethanol (avertin). A vertical incision was made in the abdominal wall, and the uterus was exposed. A small incision was made in each uterine horn at the utero-tubal junction and bilateral injury to the uterine horns was induced according to a standardized protocol; specifically, a 27-gauge needle inserted through the lumen was rotated and withdrawn 10 times. Fibrotic lesions in the endometrial tissues were evaluated by histological and collagen immunofluorescence staining as described below.

### Experimental Design

Animals in the Sham group did not undergo AS induction or PRP treatment. Animals assigned to the AS mice and PRP-treated AS group underwent AS induction without or with PRP treatment. At 7 days after AS induction (Jun et al., 2019), respectively, animals in the PRP-treated AS group were injected with 0.02 mL of PRP in the injured horn using an embryo transfer pipette, careful to fill the treated horn without invading the other horn (Figure 1).

In Experiment 1, nine mice were used to evaluate the effect of PRP on endometrial regeneration. The mice were euthanized at 14 days from experiment initiation (7 days after PRP treatment) and evaluated for cellular and molecular signs of fibrosis and regeneration. Experiments included histological and immunofluorescence staining, as well as measurement of fibrosis marker expression levels.

In Experiment 2, 10 mice were mated with healthy males at 14 days after experiment initiation (7 days after PRP treatment). On day 12 of pregnancy, the animals were sacrificed to evaluate the number and weight of IS.

In Experiment 3, 16 mice were mated with healthy males at 14 days after experiment initiation (7 days after PRP treatment). While all AS mice received injury to the bilateral uterine horns, half received no PRP treatment, whereas the other half received PRP treatment to the bilateral horns. Pregnancy outcomes included the time to conceive, live-birth rate, and litter size.

### Histological Staining

To investigate the endometrial structure, frozen uterine sections (12 μm) were fixed in 4% paraformaldehyde and stained with hematoxylin and eosin. The collagen areas were stained using Masson trichrome to evaluate fibrosis. Under Masson trichrome staining, the collagen fibers appeared stained in blue, the nuclei appeared stained in black, and the background appeared stained in red. The slides were examined using a suitable microscope (Leica Biosystems, St. Louis LLC, DieMen, Netherlands).

### Immunofluorescence Staining

To determine the expression of COL1A1 (1:200; NB600-408; Novus Biologicals) (Jun et al., 2019) after uterine damage, frozen uterine sections (12 μm) were fixed in 4% paraformaldehyde in phosphate buffered saline (PBS) for 10 min at room temperature, washed in PBS, and treated with 0.1% Triton X-100 for 10 min at room temperature and washed in PBS. Non-specific staining was blocked using protein block serum (Dako, Carpinteria, CA, United States). The sections were then incubated overnight with primary antibody at 4°C, washed in PBS, and incubated with secondary antibody (A-11001, 1:1000; Invitrogen) for 60 min at room temperature. After three washes in PBS, the sections were counterstained and mounted. Images were obtained using a suitable microscope (Carl Zeiss, Oberkochen, Germany) and analyzed using ZEN software (Carl Zeiss).

### RNA Preparation and Polymerase Chain Reaction Assays

Reverse transcription-quantitative polymerase chain reaction (RT-qPCR) and real-time RT-qPCR analyses were performed to evaluate the level of RNA expression of fibrosis-related factors (*Col1a1*, *Tgfβ1*, *Timp1*). Uteri (four to six mice per experimental group) were collected, immediately frozen in liquid nitrogen, and individually prepared for total RNA extraction. Total RNA was extracted from each uterine horn using Trizol Reagent (Ambion, Carlsbad, CA, United States) according to manufacturer’s instructions, and 2 μg of the extracted total RNA was subjected to RT using M-MLV reverse transcriptase (Promega, Madison, WI, United States) with random primers and oligo dT for cDNA synthesis. The synthesized cDNA was utilized for PCR with specific primers at optimized cycles. Subsequently, adequate primer pairs were used for PCR. Real-time RT-qPCR was performed by monitoring the increase in fluorescence of the SYBR Green dye (iQ SYBR Green Supermix; Bio-Rad, Waltham, MA, United States) using a real-time PCR detection system (Bio-Rad, Waltham, MA, United States). For between-sample comparison of transcript levels, a standard curve of cycle thresholds for several serial dilutions of a single cDNA sample was established and then used to calculate the relative abundance of each gene. The gene expression values were then normalized to the relative amounts of *rPL7* cDNA (Deng et al., 2019). All PCR assays were performed in duplicate. The identity of each amplicon was determined by the direct sequencing method.

### Statistical Analysis

All values represent means ± SD. Statistical analyses were performed using the Kruskal–Wallis one-way ANOVA test for more than two groups. Values of *p* < 0.05 were considered to denote statistical significance. GraphPad Prism ver. 5 software (GraphPad Software, La Jolla, CA, United States) was used for statistical analysis.

## Results

### Intrauterine Infusion of Human PRP Restores Endometrial Structure and Decreases Fibrosis in a Well-Established Murine Model of AS

**FIGURE 2:**
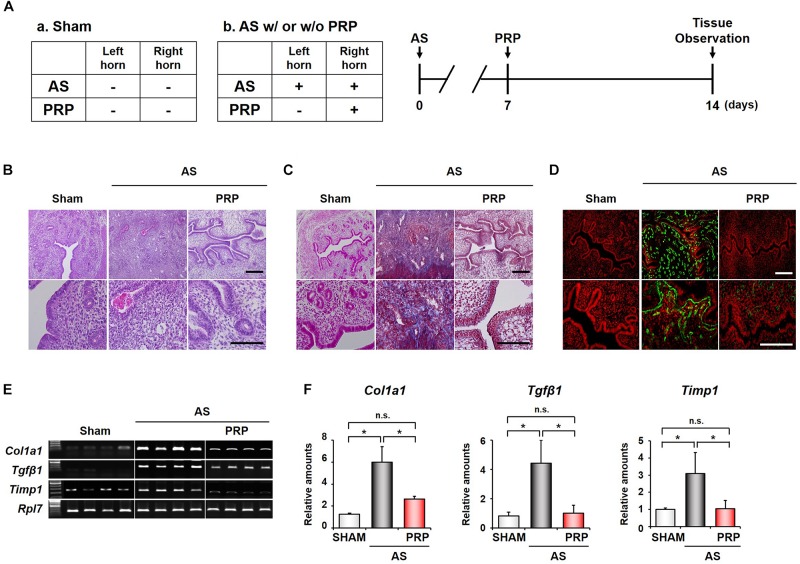
Human PRP infusion improves regeneration of the damaged endometrium and down-regulates expression of fibrosis-related factors. **(A)** Design of Experiment 1. In AS mice, PRP was injected only into the right horn at 7 days after inducing injury to the bilateral uterine horns. At 14 days, all mice were sacrificed and the uterine horns were prepared for analysis. **(B)** Hematoxylin and eosin staining of the endometrial tissues to evaluate morphologic structures in the PRP-treated horn of AS mice. **(C)** Masson’s trichrome staining to evaluate collagen deposition (blue) in the PRP-treated horns of AS mice. **(D)** Immunofluorescence staining of collagen type 1A (COL1A1) in the PRP-treated horn of AS mice. Green and red indicate COL1A1 and nuclei, respectively. **(E)** RT-qPCR and **(F)** real-time RT-qPCR analyses of expression of fibrosis-related factors in the PRP-treated horn of AS mice. Sham, *n* = 4; AS or PRP-treated AS, *n* = 5. *p < 0.01; Scale bar: 100 and 200 μm. AS, Asherman’s syndrome; PCR, polymerase chain reaction; PRP, platelet-rich plasma; RT-qPCR, reverse transcription-quantitative PCR.

In Experiment 1 (Figure 2), the adequacy of the murine AS model and the effect of human PRP treatment were assessed at 14 days after injury to the uterine horns, that is, at 7 days after PRP treatment. For this purpose, we conducted histologic examinations. In tissue sections from injured uterine horns that did not undergo PRP treatment (AS group), hematoxylin and eosin staining revealed narrow endometrial lumen lined by atrophic columnar epithelium, with degenerative changes, loss of lumen, and little stroma (Figure 2B, middle panel). However, proliferated glands and endometrial stromal cells were observed in the PRP-treated AS group (Figure 2B, right panel). Masson trichrome staining revealed that AS horns had signs of endometrial fibrosis including endometrial breakdown with trichrome-positive (blue) staining for collagen and glycosaminoglycan deposits (Figure 2C, middle panel), whereas collagen deposition was significantly lower in the PRP-treated AS horn (Figure 2C, right panel). These results indicated that PRP promoted the recovery of endometrial structure and inhibited fibrosis after uterine horn injury.

### PRP Treatment Decreases the Expression of Fibrosis-Related Factors Following Endometrial Injury

Immunofluorescence staining for COL1A1, a fibrosis-associated factor, was performed to evaluate the extent of fibrosis (Figure 2D). The expression of COL1A1 (green stain) was higher in the untreated horn of AS mice (Figure 2D, middle panel) than in the horns of Sham mice (Figure 2D, left panel), confirming that endometrial fibrosis was induced by injury to the uterine horn. However, the expression of COL1A1 was significantly lower in the PRP-treated horn (Figure 2D, right panel) than in the untreated horn of AS mice.

The expression of fibrosis-related factors including *Col1a1*, *Tgfβ1*, *and Timp1* was investigated using RT-qPCR and real-time RT-qPCR in Experiment 1 (Figures 2E,F). On RT-qPCR assay, pronounced expression of fibrosis-related factors was noted in the untreated horn of AS mice, which was significantly lower in the PRP-treated AS horn. The results of the real-time RT-qPCR assay confirmed that the expression of the three fibrosis-related factors was significantly lower in the PRP-treated horn than in the untreated horn of AS mice (*Col1a1*, 2.64 ± 0.25 vs. 5.99 ± 1.09; *Tgfβ1*, 1.01 ± 0.54 vs. 4.42 ± 1.08; *Timp1*, 1.04 ± 0.48 vs. 3.09 ± 1.01, *p* < 0.01). These results indicate that human PRP infusion helps reduce the expression of fibrosis-related factors following endometrial injury.

### PRP Treatment Enhances the Number of Implantation Sites in the Damaged Uterus

**FIGURE 3:**
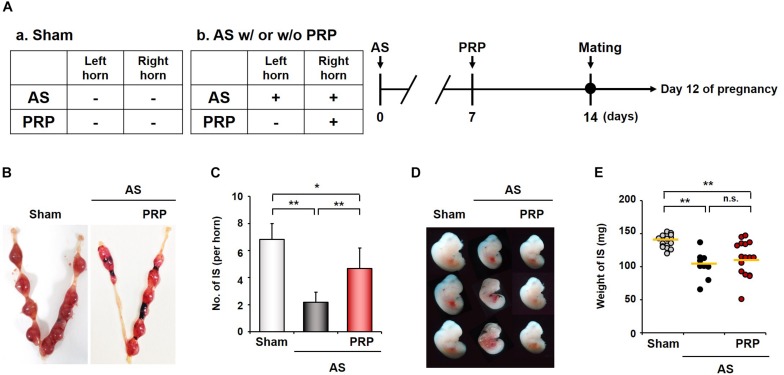
Infusion of human PRP is associated with better implantation outcomes. **(A)** Design of Experiment 2. In AS mice, injury was induced to the bilateral uterine horns; at 7 days after bilateral injury, PRP was injected only in the right uterine horn. At 14 days after experiment initiation (7 days after PRP treatment), AS mice were mated with fertile, healthy males. **(B)** Gross morphology of the implantation sites (IS) in AS mice with or without PRP. **(C)** The number of the IS in the PRP-treated horn of AS mice. **(D)** Photographs of embryos isolated from the IS on day 12 of pregnancy. **(E)** A graph for the weights of IS between the AS group and the PRP-treated AS group on day 12 of pregnancy. The horizontal yellow lines represent median values. Sham, *n* = 4; AS or PRP-treated AS, *n* = 5. *p < 0.01; **p < 0.05. AS, Asherman’s syndrome; PRP, platelet-rich plasma.

In Experiment 2 (Figure 3), the IS and embryos were investigated to assess the effects of PRP treatment on growth and development at day 12 of pregnancy (Figure 3B). The IS in Sham mice showed normal gross morphology, whereas the IS in the untreated horn of AS mice appeared abnormal and fewer in number. The number of IS was significantly higher in the PRP-treated horn than in the untreated horn of AS mice (Figure 3C; 4.67 ± 1.01 vs. 2.1 ± 0.75, *p* < 0.01), but no delay in embryo development was observed (Figure 3D). No significant differences were observed between PRP-treated and untreated horns of AS mice in terms of the distribution of weights of IS collected on day 12 of pregnancy (Figure 3E; 112 ± 31.0 vs. 102 ± 30.4 mg). These findings suggest that implantation potential can be substantially improved PRP treatment, which was reflected in a higher number of IS in AS mice treated with intrauterine PRP infusion.

### PRP Treatment Improves Live-Birth Rate in Mice With AS

**FIGURE 4:**
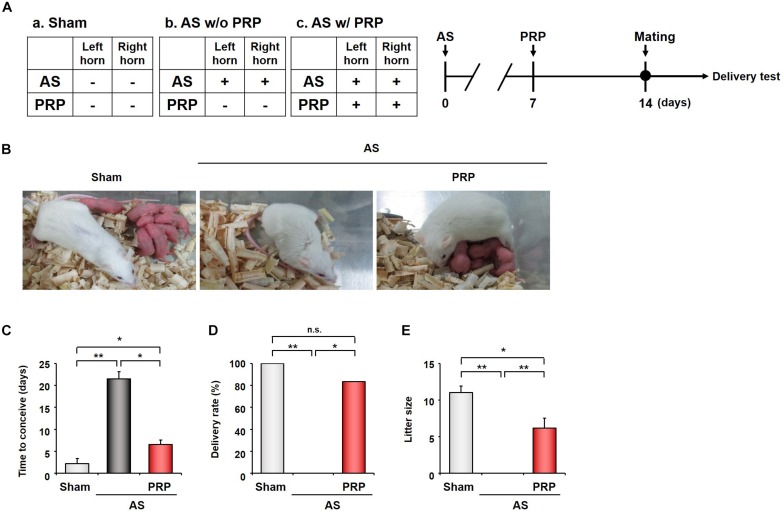
PRP treatment is associated with improved live-birth rates in mice with AS. **(A)** Design of Experiment 3. At 7 days after inducing injury to the bilateral uterine horns, half of the AS mice received PRP treatment to the bilateral horns (PRP-treated AS group), whereas the other half were followed up without PRP treatment (AS group). All mice were mated to healthy males at 14 days after experiment initiation. **(B)** Representative photographs of PRP-treated AS mice with pups after successful delivery and of AS mice which failed to deliver. **(C–E)** Therapeutic effects of PRP on the time to conceive **(C)**, delivery rate **(D)**, and litter size **(E)** in AS mice. Sham, *n* = 4; AS, *n* = 6; PRP-treated AS, *n* = 6. *p < 0.01; **p < 0.05. AS, Asherman’s syndrome; PRP, platelet-rich plasma.

In Experiment 3 (Figure 4), Sham mice took an average of 2.2 ± 1.17 days to conceive, as confirmed by vaginal plug smears conducted daily starting on the morning after the female was mated with a fertile male. However, an average of 21.5 ± 1.64 days were required for AS mice (Figure 4C), compared to only 6.5 ± 1.05 days for PRP-treated AS mice (*p* < 0.01). These results indicate that implantation was impeded by endometrial injury but could be restored by PRP treatment, though not to pre-injury levels. While all Sham mice gave birth to live pups, AS mice did not give birth to any live pups (Figure 4D), and most PRP-treated AS mice delivered healthy pups (83.3%) (*p* < 0.01). Because AS mice failed to deliver, litter size could not be compared for this group (Figure 4E). Although litter size was smaller among PRP-treated AS mice than among Sham mice (*p* < 0.01), PRP treatment clearly improved the rate of live-births since AS mice failed to deliver.

## Discussion

Several studies have suggested that PRP may be useful as an adjuvant treatment for endometrial injury (Chang et al., 2015; Farimani et al., 2017; Jang et al., 2017; Tandulwadkar et al., 2017; Zadehmodarres et al., 2017). However, these were only pilot studies or case reports that did not include a functional analysis investigating the practical effectiveness of human PRP infusion. To clarify the scientific basis for the effect of PRP treatment, we examined whether intrauterine infusion of human PRP can promote endometrial regeneration after endometrial injury and improve uterine function in a murine model of AS. The histologic and biologic analysis of uterine tissue indicated that PRP treatment restored histological structure and reduced expression of fibrosis-related factors. Furthermore, PRP treatment enhanced implantation outcomes and helped the mice carry the pregnancy to term, with delivery of viable offspring. It is important to underline that the present study establishes the theoretical basis for the clinical use of autologous PRP in women with refractory endometrium.

Adequate endometrial development plays a crucial role in the success of pregnancy. In AS, the process of endometrial regeneration is disrupted due to repeated trauma and inflammation, resulting in the formation of intrauterine adhesions (Cervello et al., 2015). Extensive evidence suggests that endometrial injury leads to poor growth of the glandular epithelium, high uterine blood flow impedance, poor angiogenesis, and the release of factors stimulating the formation of fibrotic tissue into the intrauterine environment (Shaffer, 1986; Miwa et al., 2009; Conforti et al., 2013; Mahajan and Sharma, 2016; Jang et al., 2017), which explains the association of AS with a high rate of infertility and miscarriage, poor implantation outcomes following *in vitro* fertilization, and abnormal placentation (Yu et al., 2008; Deans and Abbott, 2010; Conforti et al., 2013).

Several therapeutic options for AS aim to restore and rebuild the endometrium. Many authors have proposed extended exogenous estrogen administration to encourage the growth of residual endometrium, but there is no consensus regarding treatment duration, dosage, or method of administration (Conforti et al., 2013). Low-dose aspirin and pentoxifylline did not exhibit consistent effect in improving endometrial thickness (Mouhayar and Sharara, 2017), whereas vaginal sildenafil was reportedly effective for improving endometrial thickness and endometrial blood flow (Conforti et al., 2013). However, none of the proposed strategies have been sufficiently successful to be deemed suitable for AS treatment in the clinical setting (Yu et al., 2008; Jang et al., 2017). While the use of granulocyte-colony stimulating factor seems effective in improving endometrial thickness and possibly pregnancy rates, the studies reporting such results had small samples and did not apply a uniform treatment protocol, differing in terms of timing and dosage (Mouhayar and Sharara, 2017).

Recent interest has focused on the intrinsic capacity of endometrial regeneration, which normally occurs after menstruation and delivery because endometrial stem cells are present in both the basal and functional layers of the human endometrium (Conforti et al., 2013; Mouhayar and Sharara, 2017). Endogenous MSCs have been shown to contribute to the repair of damaged endometrium in various animal models (Alawadhi et al., 2014; Corradetti et al., 2014; Ding et al., 2014; Jing et al., 2014; Kilic et al., 2014). One study assessed the engraftment and proliferation of human BM-MSCs in a murine model of AS (Cervello et al., 2015), obtaining evidence for the clinical application of autologous BM-MSC therapy for AS in humans. A pilot cohort study of 16 patients with AS confirmed the clinical effectiveness of autologous BM-MSC therapy (Santamaria et al., 2016). However, the adoption of stem-cell therapy in clinical practice remains challenging because it requires expensive and invasive procedures, optimization of culture conditions, precise dosage of the cell infusion, and adequate means of cell transplantation (Jing et al., 2014).

Since PRP is prepared from autologous blood collected from a peripheral vein, therapy with PRP infusion is considered to safe. Moreover, the procedure of PRP preparation is convenient, affordable, and painless for patients because blood collection is relatively non-invasive. Importantly, PRP has been shown to improve the regeneration of various tissues due to release of several growth factors, cytokines, and chemokines stored in the alpha granules of platelets (Etulain et al., 2018). These molecules modulate angiogenesis, remodel the extracellular matrix, and affect the recruitment, proliferation, and differentiation of stem cells (Etulain et al., 2018). For this reason, use of PRP to promote tissue growth and repair has found application in several fields of regenerative medicine including orthopedics, dental and plastic surgery, ophthalmology, and dermatology (Tian et al., 2018).

To date, few studies have examined the potential of PRP application for restoring the damaged endometrium (Zadehmodarres et al., 2017). Such investigations reported that PRP treatment led to increased endometrial thickness (confirmed on ultrasonography), improved pregnancy rates (confirmed on clinical pregnancy tests), and improved rate of live-births (Chang et al., 2015; Farimani et al., 2017; Zadehmodarres et al., 2017; Kim et al., 2019). However, it is not clear whether the improvement was directly caused by PRP treatment. Experimental evidence remains inadequate due to lack of objective validity. One animal study demonstrated that autologous PRP treatment improved regeneration of damaged endometrium in female rats, as confirmed by histologic analysis and real-time PCR assays for the expression of factors involved in endometrial regulation and development (Jang et al., 2017). Nevertheless, that study was not able to confirm the effectiveness of human PRP and did not evaluate uterine function. In the present study, we evaluated the ability to regenerate the endometrium and the outcomes of mating in a murine model of AS with or without human PRP infusion. While our data have some limitation that hormonal profiles of donors could affect therapeutic potentials of their PRP, all PRP from different donors provided similar results, reinforcing that PRP could restore damaged endometrium. It is noteworthy that we were able to quantify both implantation and pregnancy outcomes, finding that PRP treatment significantly improved the number of IS and supported the ability to carry the pregnancy to term, leading to live-births in 83.3% of PRP-treated AS mice, whereas all AS mice failed to deliver. The time to conceive was also improved with PRP treatment. Whereas there is a practical limitation that human PRP was administered into the mouse uterus with AS, our present results provide strong evidence supporting the ability of human PRP to promote regeneration of endometrial function and to improve pregnancy outcomes.

The results suggest that PRP infusion may be a useful strategy for treating impaired endometrial function in the clinical setting. A further important task is to determine which PRP components improve which regenerative pathways, as well as to explore the underlying mechanisms. Although there was little evidence on how PRP helped endometrial regeneration, a recent study examined the effects of PRP on the biological responses of different human endometrial cells and on the expression of several pro-inflammatory cytokines, chemokines, and matrix metalloproteinases, concluding that PRP promotes endometrial regeneration at a cellular level (Aghajanova et al., 2018). Another study demonstrated that PRP up-regulates the expression of genes that play an important role in reproduction, such as estrogen receptor alpha and beta, as well as the progesterone receptor in cultured bovine endometrium (Marini et al., 2016). Our present findings add important context to these previous observations that PRP treatment affects the gene expression of pro-inflammatory factors and endometrial proliferation *in vitro*, highlighting the usefulness of *in vivo* investigations of the effect of PRP treatment in murine models of AS.

Current treatment modalities for women with impaired endometrial function are of limited efficacy; however, clinical trials to evaluate the regenerative properties of PRP have recently been initiated. While previous studies have suggested PRP may help treat impaired endometrial function in humans, we demonstrate that human PRP helps down-regulate the expression of fibrosis-related factors, restores uterine function of impaired uterine horns, and improves implantation outcomes following endometrial injury in mice, enabling full-term delivery and improving the rate of live-births. These encouraging findings establish the theoretical basis for the ability of PRP to promote endometrial regeneration and improve pregnancy outcomes, supporting the clinical application of PRP treatment in women with compromised endometrium. However, it is not clear whether the mouse model of AS recapitulates all phenotypes of human patients with AS. Thus, further studies are required for evaluating the usefulness of human PRP in the murine model of AS.

## Data Availability Statement

All datasets generated for this study are included in the article/supplementary material.

## Ethics Statement

This study was approved by the Institutional Review Board (IRB Approval No.: GCI-17-19) of the CHA Gangnam Medical Center for use of human PRP. The patients/participants provided their written informed consent to participate in this study. All experiments utilizing animals were approved by the IACUC of CHA University (Approval No.: 170088).

## Author Contributions

JK and MP contributed to the experimental procedures, analysis and interpretation of data, drafting, and revision of the manuscript. JP and W-SL contributed to the data interpretation and revision of the manuscript. HS and SL contributed to the conception and design, analysis and interpretation of data, revision, and final approval of the manuscript.

## Conflict of Interest

The authors declare that the research was conducted in the absence of any commercial or financial relationships that could be construed as a potential conflict of interest.

## References

[B2] Aghajanova L. Houshdaran S. Balayan S. Manvelyan E. Irwin J. C. Huddleston H. G. (2018). In vitro evidence that platelet-rich plasma stimulates cellular processes involved in endometrial regeneration. *J. Assist. Reprod. Genet.* 35 757–770. 10.1007/s10815-018-1130-8 29404863 PMC5984879

[B3] Alawadhi F. Du H. Cakmak H. Taylor H. S. (2014). Bone Marrow-Derived Stem Cell (BMDSC) transplantation improves fertility in a murine model of Asherman’s syndrome. *PLoS One* 9:e96662. 10.1371/journal.pone.0096662 24819371 PMC4018329

[B4] Amable P. R. Carias R. B. Teixeira M. V. da Cruz Pacheco I. Correa, do Amaral R. J. (2013). Platelet-rich plasma preparation for regenerative medicine: optimization and quantification of cytokines and growth factors. *Stem Cell Res. Ther.* 4:67. 10.1186/scrt218 23759113 PMC3706762

[B5] Burnouf T. Strunk D. Koh M. B. Schallmoser K. (2016). Human platelet lysate: replacing fetal bovine serum as a gold standard for human cell propagation? *Biomaterials* 76 371–387. 10.1016/j.biomaterials.2015.10.065 26561934

[B6] Cervello I. Gil-Sanchis C. Santamaria X. Cabanillas S. Diaz A. Faus A. (2015). Human CD133(+) bone marrow-derived stem cells promote endometrial proliferation in a murine model of Asherman syndrome. *Fertil. Steril.* 104:1552-60.e1-3. 10.1016/j.fertnstert.2015.08.032 26384164

[B7] Chan R. W. Gargett C. E. (2006). Identification of label-retaining cells in mouse endometrium. *Stem Cells* 24 1529–1538. 10.1634/stemcells.2005-0411 16456137

[B8] Chang Y. Li J. Chen Y. Wei L. Yang X. Shi Y. (2015). Autologous platelet-rich plasma promotes endometrial growth and improves pregnancy outcome during in vitro fertilization. *Int. J. Clin. Exp. Med.* 8 1286–1290. 25785127 PMC4358582

[B9] Conforti A. Alviggi C. Mollo A. De Placido G. Magos A. (2013). The management of Asherman syndrome: a review of literature. *Reprod. Biol. Endocrinol.* 11:118. 10.1186/1477-7827-11-118 24373209 PMC3880005

[B10] Corradetti B. Correani A. Romaldini A. Marini M. G. Bizzaro D. Perrini C. (2014). Amniotic membrane-derived mesenchymal cells and their conditioned media: potential candidates for uterine regenerative therapy in the horse. *PLoS One* 9:e111324. 10.1371/journal.pone.0111324 25360561 PMC4216086

[B11] De Pascale M. R. Sommese L. Casamassimi A. Napoli C. (2015). Platelet derivatives in regenerative medicine: an update. *Transfus. Med. Rev.* 29 52–61. 10.1016/j.tmrv.2014.11.001 25544600

[B12] Deans R. Abbott J. (2010). Review of intrauterine adhesions. *J. Minim. Invasive Gynecol.* 17 555–569. 10.1016/j.jmig.2010.04.016 20656564

[B13] Deng W. Yuan J. Cha J. Sun X. Bartos A. Yagita H. (2019). Endothelial cells in the decidual bed are potential therapeutic targets for preterm birth prevention. *Cell Rep.* 27:e4. 10.1016/j.celrep.2019.04.049 31067461 PMC6554729

[B14] Ding L. Li X. Sun H. Su J. Lin N. Peault B. (2014). Transplantation of bone marrow mesenchymal stem cells on collagen scaffolds for the functional regeneration of injured rat uterus. *Biomaterials* 35 4888–4900. 10.1016/j.biomaterials.2014.02.046 24680661

[B15] Etulain J. Mena H. A. Meiss R. P. Frechtel G. Gutt S. Negrotto S. (2018). An optimised protocol for platelet-rich plasma preparation to improve its angiogenic and regenerative properties. *Sci. Rep.* 8:1513. 10.1038/s41598-018-19419-6 29367608 PMC5784112

[B16] Farimani M. Poorolajal J. Rabiee S. Bahmanzadeh M. (2017). Successful pregnancy and live birth after intrauterine administration of autologous platelet-rich plasma in a woman with recurrent implantation failure: a case report. *Int. J. Reprod. Biomed.* 15 803–806. 10.29252/ijrm.15.12.803 29492478 PMC5816241

[B17] Gargett C. E. Healy D. L. (2011). Generating receptive endometrium in Asherman’s syndrome. *J. Hum. Reprod. Sci.* 4 49–52. 21772741 PMC3136070

[B18] Jang H. Y. Myoung S. M. Choe J. M. Kim T. Cheon Y. P. Kim Y. M. (2017). Effects of autologous platelet-rich plasma on regeneration of damaged endometrium in female rats. *Yonsei Med. J.* 58 1195–1203. 10.3349/ymj.2017.58.6.1195 29047244 PMC5653485

[B19] Jing Z. Qiong Z. Yonggang W. Yanping L. (2014). Rat bone marrow mesenchymal stem cells improve regeneration of thin endometrium in rat. *Fertil. Steril.* 101 587–594. 10.1016/j.fertnstert.2013.10.053 24355044

[B20] Jun S. M. Park M. Lee J. Y. Jung S. Lee J. E. Shim S. H. (2019). Single cell-derived clonally expanded mesenchymal progenitor cells from somatic cell nuclear transfer-derived pluripotent stem cells ameliorate the endometrial function in the uterus of a murine model with Asherman’s syndrome. *Cell Prolif.* 52 e12597. 10.1111/cpr.12597 30896075 PMC6536448

[B21] Kilic S. Yuksel B. Pinarli F. Albayrak A. Boztok B. Delibasi T. (2014). Effect of stem cell application on Asherman syndrome, an experimental rat model. *J. Assist. Reprod. Genet.* 31 975–982. 10.1007/s10815-014-0268-2 24974357 PMC4130935

[B22] Kim H. Shin J. E. Koo H. S. Kwon H. Choi D. H. Kim J. H. (2019). Effect of autologous platelet-rich plasma treatment on refractory thin endometrium during the frozen embryo transfer cycle: a pilot study. *Front. Endocrinol.* 10:61. 10.3389/fendo.2019.00061 30837945 PMC6382681

[B23] Lee J. W. Kwon O. H. Kim T. K. Cho Y. K. Choi K. Y. Chung H. Y. (2013). Platelet-rich plasma: quantitative assessment of growth factor levels and comparative analysis of activated and inactivated groups. *Arch. Plast. Surg.* 40 530–535. 10.5999/aps.2013.40.5.530 24086805 PMC3785585

[B24] Mahajan N. Sharma S. (2016). The endometrium in assisted reproductive technology: how thin is thin? *J. Hum. Reprod. Sci.* 9 3–8. 10.4103/0974-1208.178632 27110071 PMC4817285

[B25] Marini M. G. Perrini C. Esposti P. Corradetti B. Bizzaro D. Riccaboni P. (2016). Effects of platelet-rich plasma in a model of bovine endometrial inflammation in vitro. *Reprod. Biol. Endocrinol.* 14:58. 10.1186/s12958-016-0195-4 27619959 PMC5020481

[B26] Masuda H. Matsuzaki Y. Hiratsu E. Ono M. Nagashima T. Kajitani T. (2010). Stem cell-like properties of the endometrial side population: implication in endometrial regeneration. *PLoS One* 5:e10387. 10.1371/journal.pone.0010387 20442847 PMC2860997

[B27] Miwa I. Tamura H. Takasaki A. Yamagata Y. Shimamura K. Sugino N. (2009). Pathophysiologic features of “thin” endometrium. *Fertil. Steril.* 91 998–1004. 10.1016/j.fertnstert.2008.01.029 18328483

[B28] Mouhayar Y. Sharara F. I. (2017). G-CSF and stem cell therapy for the treatment of refractory thin lining in assisted reproductive technology. *J. Assist. Reprod. Genet.* 34 831–837. 10.1007/s10815-017-0922-6 28405864 PMC5476539

[B29] Santamaria X. Cabanillas S. Cervello I. Arbona C. Raga F. Ferro J. (2016). Autologous cell therapy with CD133+ bone marrow-derived stem cells for refractory Asherman’s syndrome and endometrial atrophy: a pilot cohort study. *Hum. Reprod.* 31 1087–1096. 10.1093/humrep/dew042 27005892

[B30] Senturk L. M. Erel C. T. (2008). Thin endometrium in assisted reproductive technology. *Curr. Opin. Obstet. Gynecol.* 20 221–228. 10.1097/GCO.0b013e328302143c 18460935

[B31] Shaffer W. (1986). Role of uterine adhesions in the cause of multiple pregnancy losses. *Clin. Obstet. Gynecol.* 29 912–924. 10.1097/00003081-198612000-00016 3545591

[B32] Tandulwadkar S. R. Naralkar M. V. Surana A. D. Selvakarthick M. Kharat A. H. (2017). Autologous intrauterine platelet-rich plasma instillation for suboptimal endometrium in frozen embryo transfer cycles: a pilot study. *J. Hum. Reprod. Sci.* 10 208–212. 10.4103/jhrs.JHRS_28_17 29142450 PMC5672727

[B33] Tian J. Lei X. X. Xuan L. Tang J. B. Cheng B. (2018). The effects of aging, diabetes mellitus, and antiplatelet drugs on growth factors and anti-aging proteins in platelet-rich plasma. *Platelets* 30 773–792. 10.1080/09537104.2018.1514110 30252623

[B34] Yu D. Wong Y. M. Cheong Y. Xia E. Li T. C. (2008). Asherman syndrome–one century later. *Fertil. Steril.* 89 759–779. 10.1016/j.fertnstert.2008.02.096 18406834

[B35] Zadehmodarres S. Salehpour S. Saharkhiz N. Nazari L. (2017). Treatment of thin endometrium with autologous platelet-rich plasma: a pilot study. *JBRA Assist. Reprod.* 21 54–56. 10.5935/1518-0557.20170013 28333034 PMC5365202

